# Competitive Ligand-Induced Recruitment of Coactivators to Specific PPARα/δ/γ Ligand-Binding Domains Revealed by Dual-Emission FRET and X-Ray Diffraction of Cocrystals

**DOI:** 10.3390/antiox14040494

**Published:** 2025-04-20

**Authors:** Shotaro Kamata, Akihiro Honda, Sayaka Yashiro, Chihiro Kaneko, Yuna Komori, Ayumi Shimamura, Risa Masuda, Takuji Oyama, Isao Ishii

**Affiliations:** 1 Department of Health Chemistry, Showa Pharmaceutical University, Machida, Tokyo 194-8543, Japan; 2 Faculty of Life and Environmental Sciences, University of Yamanashi, Kofu, Yamanashi 400-8510, Japan

**Keywords:** coregulator, corepressor, AF-2 helix 12, peroxisome proliferator-activated receptor, fluorescence resonance energy transfer, transcription factor, X-ray crystallography

## Abstract

Peroxisome proliferator-activated receptors (PPARs), composed of the α/δ/γ subtypes, are ligand-activated nuclear receptors/transcription factors that sense endogenous fatty acids or therapeutic drugs to regulate lipid/glucose metabolism and oxidative stress. PPAR forms a multiprotein complex with a retinoid X receptor and corepressor complex in an unliganded/inactive state, and ligand binding induces the replacement of the corepressor complex with the coactivator complex to initiate the transcription of various genes, including the metabolic and antioxidant ones. We investigated the processes by which the corepressor is replaced with the coactivator or in which two coactivators compete for the PPARα/δ/γ-ligand-binding domains (LBDs) using single- and dual-emission fluorescence resonance energy transfer (FRET) assays. Single-FRET revealed that the respective PPARα/δ/γ-selective agonists (pemafibrate, seladelpar, and pioglitazone) induced the dissociation of the two corepressor peptides, NCoR1 and NCoR2, from the PPARα/δ/γ-LBDs and the recruitment of the two coactivator peptides, CBP and TRAP220. Meanwhile, dual-FRET demonstrated that these processes are simultaneous and that the four coactivator peptides, CBP, TRAP220, PGC1α, and SRC1, were competitively recruited to the PPARα/δ/γ-LBDs with different preferences upon ligand activation. Furthermore, the five newly obtained cocrystal structures using X-ray diffraction, PPARα-LBDs–NCoR2/CBP/TRAP220/PGC1α and PPARγ-LBD–NCoR2, were co-analyzed with those from our previous studies. This illustrates that these coactivators bound to the same PPARα-LBD loci via their consensus LXXLL motifs in the liganded state; that NCoR1/NCoR2 corepressors bound to the same loci via the IXXXL sequences within their consensus LXXXIXXXL motifs in the unliganded state; and that ligand activation induced AF-2 helix 12 formation that interfered with corepressor binding and created a binding space for the coactivator. These PPARα/γ-related biochemical and physicochemical findings highlight the coregulator dynamics on limited PPARα/δ/γ-LBDs loci.

## 1. Introduction

Peroxisome proliferator-activated receptors (PPARs) belong to the nuclear receptor (NR) superfamily and are ligand-activated transcription factors that sense endogenous free fatty acids or therapeutic drugs [1]. In mammals, three cognate subtypes with distinct tissue distributions and functions, PPARα(/NR1C1), PPARδ(/PPARβ/NR1C2), and PPARγ(/NR1C3), have been identified [2]. PPARα regulates the adaptative response to fasting by controlling fatty acid transport, peroxisomal/mitochondrial oxidation, and ketogenesis [1,2,3,4]. The fibrate-type PPARα agonists, including bezafibrate, ciprofibrate, fenofibrate, clofibrate, and pemafibrate, have been utilized to treat hypertriglyceridemia for decades. Meanwhile, PPARγ is the master regulator of adipogenesis that induces adipose differentiation and increases fatty acid uptake/storage, thereby improving insulin sensitivity [1,4]. The PPARγ agonist, pioglitazone has been employed for treating type 2 diabetes since 1999. Fenofibrate and pioglitazone were the 88th and 120th most commonly prescribed medications, with 1.96 and 1.16 million referrals, respectively, in the United States in 2022 [5]. In general, PPARα and PPARγ have contrasting metabolic effects as well as shared functions against inflammation [4]. The (patho)physiological roles of PPARδ are understood less, perhaps because the PPARδ-selective agonists were not available clinically until the accelerated approval of seladelpar on 8/14/2024 for the treatment of primary biliary cholangitis [6]. PPARδ activation improved both dyslipidemia and insulin sensitivity in rodent models, and PPARδ may be different or similar to PPARα/γ [3]. PPARδ-selective agonists, such as seladelpar, are expected to be effective against dyslipidemia, diabetes, obesity, liver diseases, and sporadic inclusion body myositis [6]. Importantly, most PPAR agonists can act on two or three of the PPAR subtypes as dual/pan agonists [7,8].

PPAR occurs in the nucleus, forms a heterodimer with the retinoid X receptor (RXR), and binds to the *cis*-acting regulatory region, which contains PPAR-response elements (PPREs) upstream of the target genes [9]. The unliganded PPAR/RXR is bound to the multiprotein corepressor complex that works coherently with histone deacetylases (HDACs) to silence the transcription of target genes [10,11,12,13]. Synthetic PPAR antagonists stabilize such inactive states [14]. The activation of PPAR and RXR by their ligands [15,16] induces a conformational change in PPAR, thereby releasing the corepressor complex and recruiting the coactivator complex to the promoters of target genes to initiate their transcription [12,17,18]. These genes control energy homeostasis [11] and exert anti-oxidant and anti-inflammatory impacts by suppressing NF-kB and reactive oxygen species, but upregulating the levels of antioxidant enzymes [19,20]. By employing single-emission fluorescence resonance energy transfer (FRET) assay, we have recently shown that four coactivator peptides, cyclic AMP responsive element binding protein (CREB)-binding protein (CBP); thyroid hormone receptor-associated protein 220 (TRAP220); PPARγ coactivator-1α (PGC1α); and steroid receptor coactivator 1 (SRC1) were differentially recruited by varying combinations of PPARα/δ/γ-ligand-binding domains (LBDs) and eight PPAR-selective/dual/pan agonists [21]. We also revealed the structures of 11 PPARα/γ-LBDs–ligand–SRC1 complexes by X-ray crystallography [22,23,24] although others’ and our efforts to obtain the PPARδ-LBDs–any coactivator/corepressor cocrystals were all unsuccessful. Additionally, the structures of 27 PPARα/γ-LBDs–(antagonist–)corepressor complexes have been reported since the first one, which was that of the PPARα-LBD–GW6471–NCoR2 (nuclear receptor corepressor 2; also known as SMRT; PDB ID: **1KKQ**) [14]. However, the structural information about the unliganded PPARα/δ/γ-LBDs–corepressor complexes is far from sufficient.

In the present study, we examined the competitive binding between the corepressor and coactivator or the two coactivators with the PPARα/δ/γ-LBDs utilizing a dual-(emission) FRET assay. Additionally, we obtained more PPARα/δ/γ-LBDs–coactivator/corepressor cocrystal structures employing X-ray diffraction to illuminate the coregulator binding loci within the PPARα/δ/γ-LBDs.

## 2. Materials and Methods

### 2.1. Recombinant PPARα/δ/γ-LBD Expression and Purification

Human PPARα-LBD (amino acids [AAs] 200–468), PPARδ-LBD (AAs 170–441), and PPARγ-LBD (AAs 203–477 in isoform 1) were expressed in Rosetta (DE3) pLysS competent cells (Merck KGaA [Novagen], Darmstadt, Germany) as amino-terminal His-tagged proteins using a pET28a vector (Novagen) [22,25] and were utilized for both biochemical and physicochemical analyses. The three-step chromatography using a cobalt-based immobilized metal affinity column containing a TALON metal affinity resin (TaKaRa Bio, Shiga, Japan), a HiTrap Q anion-exchange column (GE Healthcare, Chicago, IL, USA), and a HiLoad 16/600 Superdex 75 pg gel-filtration column (GE Healthcare) was utilized to purify each recombinant protein as previously described [22,25]. For structural analyses of the cocrystals, His-tags were cleaved with thrombin protease (Nacalai Tesque, Kyoto, Japan) after affinity chromatography.

### 2.2. Single- and Dual-FRET Assays

Proximal interactions between His-tagged hPPARα/δ/γ-LBD proteins and two biotin-labeled corepressor peptides with the consensus α-helical Leu-X-X-X-Ile-X-X-X-Leu (LXXXIXXXL, X: any amino acid) motifs [14] or four biotin (or fluorescein isothiocyanate [FITC]-Ahx)-labeled coactivator peptides with the consensus α-helical Leu-X-X-Leu-Leu (LXXLL) motifs [14] were investigated utilizing a LANCE Ultra TR-FRET assay (PerkinElmer, Shelton, CT, USA) [21,22,23,24,26]. The peptides, NCoR1 corepressor (biotin-ADPASNLGLEDIIRKALMGSF [AAs 2255–2275], which corresponds to N-CoR ID2 in [27]), NCoR2 corepressor (biotin-EHASTNMGLEAIIRKALMGKY [AAs 2321–2341], which corresponds to SMRT ID-C in [28]), CBP coactivator (biotin (or FITC-Ahx)-SGNLVPDAASKHKQLSELLRGGSGS [AAs 56–80], which corresponds to CBP in [29]), PGC1α coactivator (biotin-EAEEPSLLKKLLLAPANTQ [AAs 137–155], which corresponds to PGC-1 in [29]), SRC1 coactivator (biotin-CPSSHSSLTERHKILHRLLQEGSPS [AAs 676–700], which corresponds to SRC-1 M2 in [29]), and TRAP220 coactivator (biotin (or FITC-Ahx)-PVSSMAGNTKNHPMLMNLLKDNPAQ [AAs 631–655], which corresponds to the above coactivator 25 amino acid sequences), were synthesized by GenScript, Tokyo, Japan. A 9.5 µL aliquot of 400 nM PPARα/δ/γ-LBD in buffer A (20 mM HEPES [pH 7.4], 150 mM NaCl, 1 mM EDTA, 1 mM dithiothreitol, 0.005% Tween 20, 0.1% fatty acid-free bovine serum (albumin), 0.5 µL of a 100× ligand solution in DMSO, and 5 µL of 1 µM biotin-coactivator peptide in buffer A were mixed in single well of a 384-well low-volume, white-round-bottom, polystyrene nonbinding surface microplate (Cat no. 4513, Corning, Charlotte, NC, USA). Next, 5 µL of 2 nM terbium (Tb) cryptate-labeled anti-6×His antibody (Revvity, Waltham, WA, USA)/80 nM ULight-Streptavidin (Revvity) was added to each well and the microplate was dark-incubated at room temperature for 2 h. FRET signals were measured at an integration time of 200 µs and a delay time of 100 µs, utilizing single excitation (340/12) and three emission (490/12, 520/12, and 665/12) filters for the detection of Tb cryptate, FITC-FRET, and ULight-FRET signals, respectively on a Varioskan Flash spectral scanning multimode reader (Thermo Fisher Scientific, Waltham, MA, USA). The 520/490 and 665/490 signal ratios were calculated and normalized to those of the negative controls obtained with the solvent, 1% DMSO. Nonlinear fitting was performed and EC_50_ was calculated using the Prism 9 software (GraphPad, Boston, MA, USA). Pemafibrate was kindly provided by Kowa Company, Ltd. (Tokyo, Japan). Seladelpar and pioglitazone were purchased from ChemScene LLC. (Monmouth Junction, NJ, USA) and Cayman Chemical (Ann Arbor, MI, USA), respectively.

### 2.3. Cocrystallization of PPARα/γ-LBD with Coregulator Peptides

PPARα-LBD–NCoR2 (TNMGLEAIIRKALMGKYDQWEE) was cocrystallized in hanging-drop mixtures containing 0.5 µL PPARα-LBD (20 mg/mL in buffer B: 20 mM Tris-HCl [pH 8.0], 150 mM NaCl, 1 mM Tris 2-carboxyethylphosphine [TCEP]-HCl, and 10% glycerol), 0.5 µL NCoR2 peptide (2 mM in buffer B), and 1 µL reservoir solution (0.1 M HEPES [pH 7.0], 14% PEG8000, and 0.2 M MgCl_2_) at 20 °C for several weeks. PPARα-LBD–intrinsic fatty acid (iFA; [22])–CBP (DAASKHKQLSELLRGGSGS) was cocrystallized in hanging-drop mixtures containing 0.5 µL PPARα-LBD (20 mg/mL in buffer B), 0.5 µL CBP peptide (2 mM in buffer B), and 1 µL reservoir solution (0.1 M Tris [pH 8.5], 30% PEG4000, and 0.2 M sodium acetate trihydrate) using crushed PPARα-LBD–iFA cocrystals as crystal cores [22,25] at 4 °C for several weeks. PPARα-LBD–GW7647–TRAP220 (NTKNHPMLMNLLKDNPAQD) was cocrystallized in hanging-drop mixtures containing 0.5 µL PPARα-LBD (20 mg/mL in buffer B), 0.5 µL TRAP220 peptide (2 mM in buffer B), GW7647 (2 mM in buffer B), and 1 µL reservoir solution (0.1 M Tris [pH 8.5], 25% PEG3350, and 0.2 M ammonium acetate) utilizing crushed PPARα-LBD–iFA cocrystals as crystal cores at 4 °C for several weeks. PPARα-LBD–iFA/GW7647–PGC1α (EAEEPSLLKKLLLAP) was cocrystallized in hanging-drop mixtures of 0.5 µL PPARα-LBD (20 mg/mL in buffer B), 0.5 µL PGC1α peptide (2 mM in buffer B), without/with GW7647 (2 mM in buffer B), and 1 µL reservoir solution (0.1 M HEPES [pH 7.0]/25% PEG3350) using PPARα-LBD–iFA cocrystal crushes as crystal cores at 4 °C for several weeks.

PPARγ-LBD–NCoR2 was cocrystallized in hanging-drop mixtures containing 0.5 µL PPARγ-LBD (20 mg/mL in buffer C), 0.5 µL NCoR2 peptide (2 mM in buffer C), and 1 µL reservoir solution (0.1M Tris [pH 8.5], 30% PEG8000, and 0.2 M ammonium sulfate) at 20 °C for several weeks.

All cocrystals obtained were briefly soaked in a cryoprotection buffer (each reservoir solution + 20% (*w*/*v*) glycerol for PPARα-LBD crystals and 30% glycerol for PPARγ-LBD crystals). Subsequently, these were flash-frozen in a stream of liquid N_2_ until X-ray diffraction was conducted.

### 2.4. X-Ray Diffraction: Data Collection and Model Refinement

Datasets were collected by a BL-5A, BL-17A, or AR-NW12A beamline at the Photon Factory (Ibaraki, Japan) utilizing a synchrotron radiation of 1.0 Å. Diffraction data were collected at an oscillation of 0.1° per frame; a total of 1800 frames (180°) were recorded employing 1.0-Å X-ray crystallography [22,23,24,26]. Data processing and scaling were performed using the XDS X-ray detector software (ver. Mar 15, 2019) [30] and the AIMLESS software (ver. 0.5.21) [31], respectively. Resolution cutoff values (*R*merge < 0.5, *R*pim < 0.3, and completeness > 0.9) were set by the maximal resolution shell. All structures were determined using the molecular replacement tool in the Phaser crystallographic software (ver. 2.7.6) [32] with the following Protein Data Bank (PDB) IDs: **1KKQ** for PPARα-LBD/NCoR2, **7BQ1** for PPARα-LBD/coactivators, and **6MS7** for PPARγ-LBD as the search model. Refinement employed the iterative cycles of the model adjustment using two programs: COOT (ver. 0.8.2) [33] and PHENIX (ver. 1.11.1-2575-000) [34]. The structures were constructed with PyMOL programs (ver. 1.8.x). All data collection and refinement statistics are summarized in Supplementary Materials Table S1 and deposited in the PDB with accession numbers: **9IWJ**, **9IWK**, **9IWL**, **9IWM**, **9IWN**, and **9IWO**.

## 3. Results

### 3.1. Dual-FRET Assay to Monitor Coregulator Attachment

Single-emission FRET is widely used to evaluate ligand-induced coactivator recruitment to NRs with high sensitivity. Furthermore, multiplex FRET can be achieved by using Tb donor and certain fluorescent acceptors [35]. The ligand-dependent dissociation of the NCoR2 peptide and the association of the CBP1 peptide from/with PPARα/γ-LBDs was simultaneously monitored by dual-FRET, employing CS124-TTHA-Tb [36], fluorescein, and Alexa Fluor 633 in 2007 [37]. However, follow-up PPAR research is missing. This study utilized a combination of Tb cryptate [38], FITC, and ULight fluorophore, partially based on the LanthaScreen TR-FRET gamma coactivator assay kit (Thermo Fisher Scientific), to investigate the PPARγ-LBD–coregulator interactions. PPARγ-LBD recombinant proteins were selected for the setup as they were mainly prepared as apo (unliganded) forms. This is distinct from PPARα/δ-LBDs, which substantially contain endogenous fatty acids during recombinant protein preparation [22].

**Figure 1:**
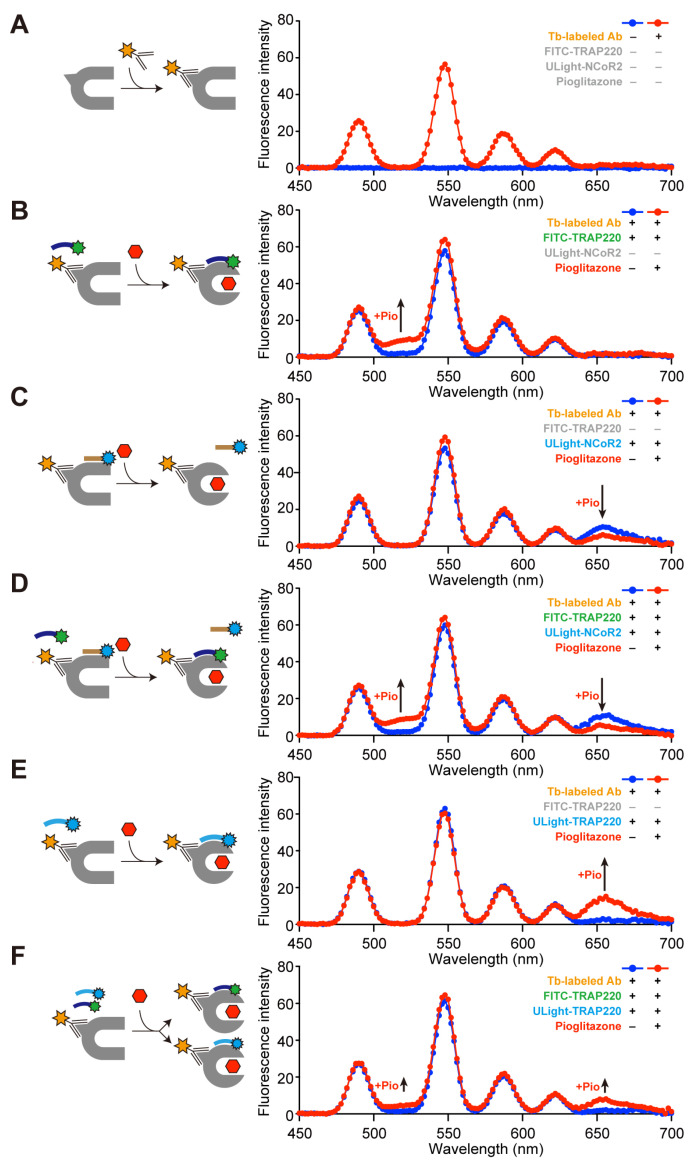
Single- and dual-FRET assays detected agonist-induced coactivator recruitment and corepressor removal from the PPARα-LBD. (**A**) Baseline fluorescence (450–700 nm) in the presence (red)/absence (blue) of the terbium (Tb)-labeled antibody. (**B**) Pioglitazone upregulated the 520 nm fluorescence by recruiting the FITC-TRAP220 coactivator as indicated by single-FRET. (**C**) Pioglitazone downregulated the 665 nm fluorescence by removing the ULight-NCoR2 corepressor as indicated by single-FRET. (**D**) Pioglitazone upregulated the 520 nm fluorescence while suppressing the 665 nm fluorescence by removing ULight-NCoR2 and recruiting FITC-TRAP220 ascertained by employing dual-FRET. (**E**) Pioglitazone elevated the 665 nm fluorescence by recruiting the ULight-TRAP220 coactivator determined using single-FRET. (**F**) Pioglitazone upregulated the 520 nm and 665 nm fluorescence by the competitive recruitment of FITC-TRAP220 and ULight-TRAP220, respectively, indicated by dual-FRET. The fluorescent intensity was monitored at every 2 nm.

Upon excitation at 340 nm, the emission spectrum of Tb conjugated with PPARγ-LBD via the His-tag was characterized based on four distinct bands, centered at 490, 546, 583, and 620 nm; emission was negligible between and beyond these peaks (Figure 1A). Although the addition of FITC-TRAP220 to Tb did not induce any spectral alterations, pioglitazone increased the emission centered at 520 nm where Tb emission was negligible (Figure 1B). Meanwhile, the supplementation of Tb with the ULight-NCoR2 corepressor enhanced the emission centered at 665 nm, where the Tb emission was negligible, but was counteracted by pioglitazone (Figure 1C). The co-addition of Tb with FITC-TRAP220 and ULight-NCoR2 elevated the 520 nm emission and reduced the 665 nm emission (Figure 1D). Likewise, the introduction of ULight-TRAP220 to Tb did not induce any spectral changes, but pioglitazone increased the 665 nm emission (Figure 1E). Finally, the coaddition of Tb with FITC-TRAP220 and ULight-TRAP220 enhanced the 520 and 665 nm emissions, although their peaks were about half of those with a single addition (Figure 1F). These results indicate that dual-FRET can simultaneously detect the replacement of the corepressor (peptide) with the coactivator (peptide) or the binding of the competitive coactivators (peptides) to the PPAR-LBD.

### 3.2. Simultaneous Corepressor Dissociation and Coactivator Recruitment Revealed by the Dual-FRET Assay

**Figure 2:**
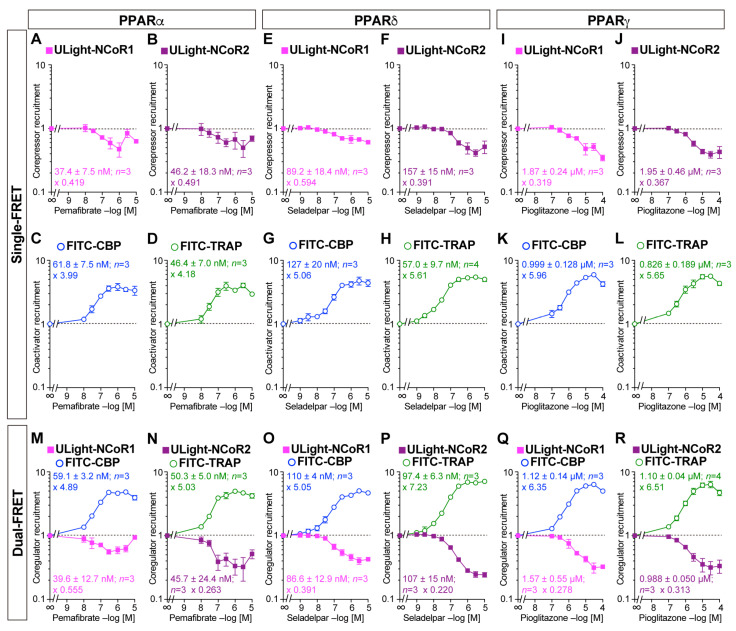
Single-FRET assay detected ligand-induced corepressor dissociation or coactivator recruitment and dual-FRET assay detected both concerning PPARα/δ/γ-LBD. (**A**–**L**) Single-FRET. Ligand (pemafibrate for PPARα, (**A**–**D**); seladelpar for PPARδ, (**E**–**H**); and pioglitazone for PPARγ, (**I**–**L**)) concentration-dependent dissociation of ULight-NCoR1 (**A**,**E**,**I**) or ULight-NCoR2 (**B**,**F**,**J**) corepressors, and recruitment of FITC-CBP (**C**,**G**,**K**) or FITC-TRAP (**D**,**H**,**L**) coactivators. (**M**–**R**) Dual-FRET. Ligand concentration-dependent dissociation of the corepressor and recruitment of the coactivator (ULight-NCoR1/FITC-CBP set for (**M**,**O**,**Q**); ULight-NCoR2/FITC-TRAP set for (**N**,**P**,**R**)) regarding PPARα/δ/γ-LBD. Data are expressed as the means ± SE of 3–4 experiments with duplicate samples. The EC_50_/IC_50_ values obtained and the maximum fold-change responses against the vehicle (no ligand) are indicated.

We first independently investigated corepressor dissociation and coactivator recruitment with PPARα/δ/γ-LBDs induced by their respective ligands, pemafibrate [39], seladelpar [40], and pioglitazone, using single-FRET [41]. Each ligand induced the concentration-dependent dissociation of the ULight-NCoR1 or ULight-NCoR2 peptides from PPARα/δ/γ-LBDs as well as the concentration-dependent recruitment of FITC-CBP or FITC-TRAP peptides (Figure 2A–L). The IC_50_ values for corepressor dissociation were almost equal to the EC_50_ values for coactivator recruitment. Each ligand at the maximum induced 0.391–0.594-fold changes (in the vehicle control) in corepressor dissociation, whereas it induced 3.99–5.96-fold alterations in coactivator recruitment.

Next, dual-FRET was employed to simultaneously detect ligand-induced corepressor dissociation and coactivator recruitment. Pemafibrate had induced ULight-NCoR1 corepressor dissociation as well as FITC-CBP coactivator recruitment with IC_50_/EC_50_ values and fold changes similar to those in single-FRET (Figure 2M). The same was true for ULight-NCoR2/FITC-TRAP with PPARα-LBD (Figure 2N), ULight-NCoR1/FITC-CBP with PPARδ-LBD (Figure 2O), ULight-NCoR2/FITC-TRAP with PPARδ-LBD (Figure 2P), ULight-NCoR1/FITC-CBP with PPARγ-LBD (Figure 2Q), and ULight-NCoR2/FITC-TRAP with PPARγ-LBD (Figure 2R). These findings suggest the simultaneous corepressor dissociation + coactivator association with all PPARα/δ/γ-LBDs upon ligand activation.

### 3.3. Competitive Coactivator Recruitment Revealed by Dual-FRET Assay

We recently reported the differential recruitment, in both efficacy and efficiency, of four coactivators, CBP, TRAP220, PGC1α, and SRC1 with PPARα/δ/γ-LBDs by employing eight PPAR agonists [21]. As each PPARα/δ/γ-LBD can accept (bind to) only one LXXLL motif of the coactivator, such differential recruitment could alter the regulation of target gene transcription [21]. Using dual-FRET, we next investigated competitive coactivator recruitment.

**Figure 3:**
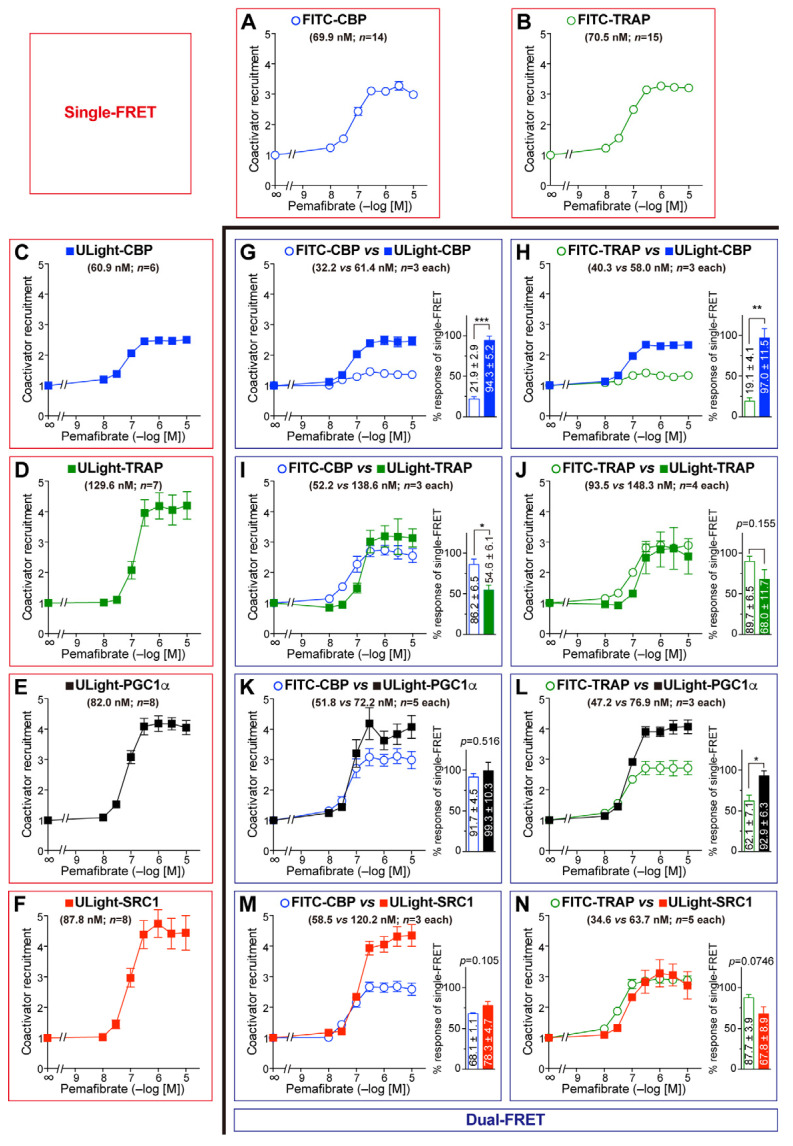
Pemafibrate-induced competitive coactivator recruitment to the PPARα-LBD. (**A**–**F**) Single-FRET assay detected the recruitment of FITC-CBP (**A**), FITC-TRAP220 (**B**), ULight-CBP (**C**), ULight-TRAP220 (**D**), ULight-PGC1α (**E**), and ULight-SRC1 (**F**). (**G**–**N**) Dual-FRET assay ascertained the competitive recruitment of FITC-CBP/ULight-CBP (**G**), FITC-TRAP220/ULight-CBP (**H**), FITC-CBP/ULight-TRAP220 (**I**), FITC-TRAP220/ULight-TRAP220 (**J**), FITC-CBP/ULight-PGC1α (**K**), FITC-TRAP220/ULight-PGC1α (**L**), FITC-CBP/ULight-SRC1 (**M**), and FITC-TRAP220/ULight-SRC1 (**N**). The EC_50_ values and sample numbers (*n*) are indicated in parentheses. The percent maximal responses (as fold-change responses against the vehicle) of the coactivator recruitment indicated by dual-FRET against the maximal responses determined by single-FRET (in **A**–**F**) are shown as bar graphs. Differences in the maximal responses were significant at * *p* < 0.05, ** *p* < 0.01, and *** *p* < 0.001, obtained via unpaired Student *t*-tests.

First, we examined all eight combination sets between FITC-CBP/TRAP220 and ULight-CBP/TRAP220/PGC1α/SRC1 with the PPARα-LBD activated by pemafibrate (Figure 3). Notably, FITC-CBP and ULight-CBP were variably recruited (Figure 3G), while FITC-TRAP and ULight-TRAP were employed similarly (Figure 3J), probably due to the different effects of the fluorescent labeling of each peptide. When the maximal procurement of FITC-CBP was considered 100, those of ULight-CBP/TRAP220/PGC1α/SRC1 were 431, 63.3, 108, and 115, respectively (Figure 3G, I, K, and M, respectively). When the peak recruitment of FITC-TRAP was assumed as 100, those of ULight-CBP/TRAP220/PGC1α/SRC1 were 508, 75.8, 150, and 77, respectively (Figure 3H, J, L, and N, respectively). In summary, the coactivator preference of PPARα-LBD was presumed to be CBP >> PGC1α = SRC1 > TRAP220. This is generally similar to the reverse EC_50_ orders in single-FRET; namely, CBP (60.9 nM) < PGC1α (82.0 nM) = SRC1 (87.8 nM) < TRAP220 (129.6 nM) (Figure 3C–F).

**Figure 4:**
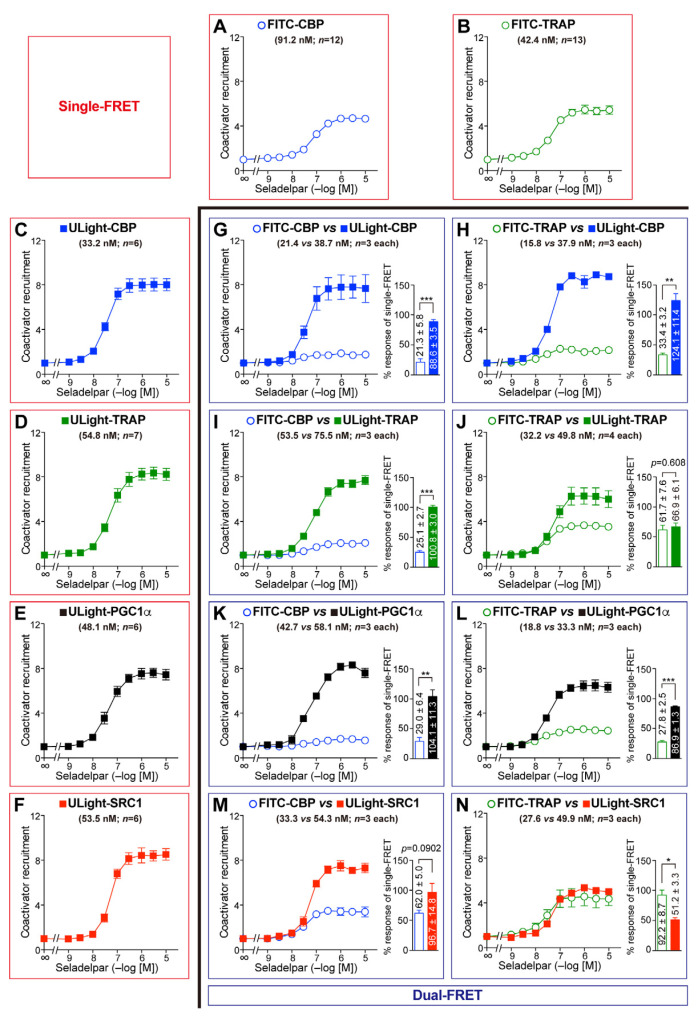
Seladelpar-induced competitive coactivator recruitment to the PPARδ-LBD. (**A**–**F**) Single-FRET assay detected the recruitment of FITC-CBP (**A**), FITC-TRAP220 (**B**), ULight-CBP (**C**), ULight-TRAP220 (**D**), ULight-PGC1α (**E**), and ULight-SRC1 (**F**). (**G**–**N**) Dual-FRET assay ascertained the competitive recruitment of FITC-CBP/ULight-CBP (**G**), FITC-TRAP220/ULight-CBP (**H**), FITC-CBP/ULight-TRAP220 (**I**), FITC-TRAP220/ULight-TRAP220 (**J**), FITC-CBP/ULight-PGC1α (**K**), FITC-TRAP220/ULight-PGC1α (**L**), FITC-CBP/ULight-SRC1 (**M**), and FITC-TRAP220/ULight-SRC1 (**N**). The EC_50_ values and sample numbers (*n*) are represented in parentheses. The percent maximal responses (as fold-change responses against the vehicle) of the coactivator recruitment determined using dual-FRET against the maximal responses obtained utilizing single-FRET (in **A**–**F**) are shown as bar graphs. Differences in the maximal responses were significant at * *p* < 0.05, ** *p* < 0.01, and *** *p* < 0.001, obtained via unpaired Student *t*-tests.

Next, we examined all of the eight combination sets with the PPARδ-LBD activated by seladelpar (Figure 4). Similarly, FITC-CBP and ULight-CBP were variably recruited (Figure 4G). In contrast, FITC-TRAP and ULight-TRAP were similarly employed (Figure 4J). When the maximal procurement of FITC-CBP was considered 100, those of ULight-CBP/TRAP220/PGC1α/SRC1 were 508, 402, 359, and 156, respectively (Figure 4G, I, K, and M, respectively). When the peak recruitment of FITC-TRAP was considered 100, those of ULight-CBP/TRAP220/PGC1α/SRC1 were 372, 108, 313, and 55.5, respectively (Figure 4H, J, L, and N, respectively). Taken together, the coactivator preference of PPARα-LBD was assumed to be CBP > PGC1α ≥ TRAP220 > SRC1. this is generally similar to the reverse EC_50_ orders in single-FRET; namely, CBP (33.2 nM) < PGC1α (48.1 nM) ≤ SRC1 (53.5 nM) = TRAP220 (54.8 nM) (Figure 4C–F).

**Figure 5:**
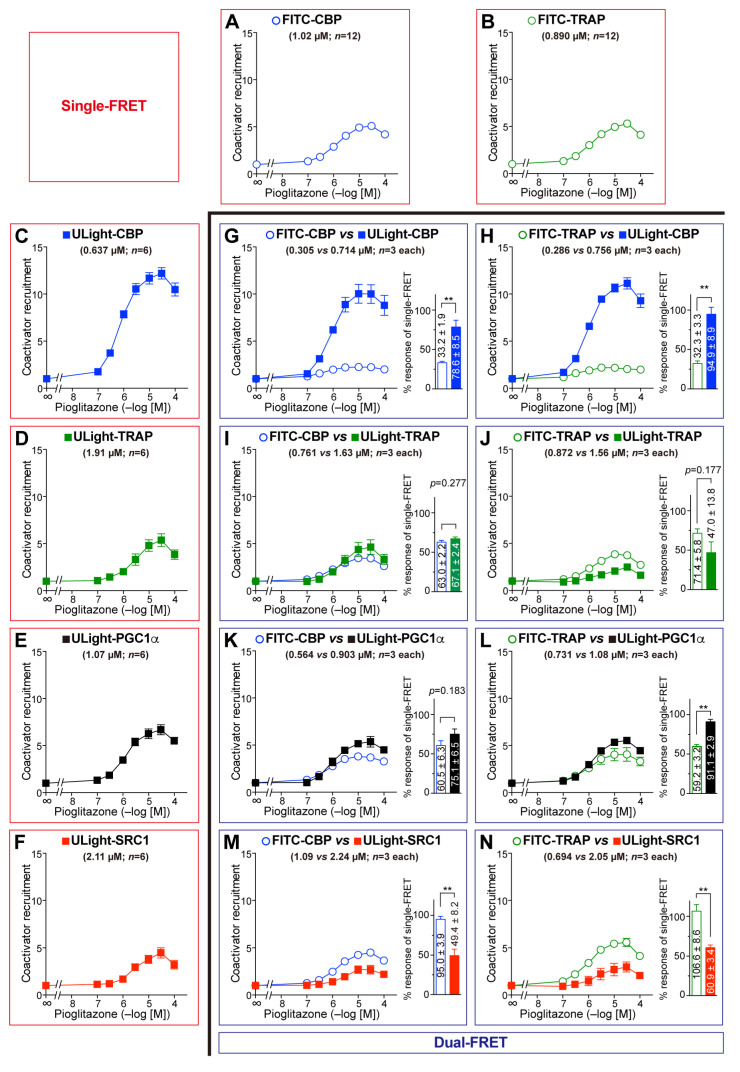
Pioglitazone-induced competitive coactivator recruitment to the PPARγ-LBD. (**A**–**F**) Single-FRET assay detected the recruitment of FITC-CBP (**A**), FITC-TRAP220 (**B**), ULight-CBP (**C**), ULight-TRAP220 (**D**), ULight-PGC1α (**E**), and ULight-SRC1 (**F**). (**G**–**N**) Dual-FRET assay identified the competitive recruitment of FITC-CBP/ULight-CBP (**G**), FITC-TRAP220/ULight-CBP (**H**), FITC-CBP/ULight-TRAP220 (**I**), FITC-TRAP220/ULight-TRAP220 (**J**), FITC-CBP/ULight-PGC1α (**K**), FITC-TRAP220/ULight-PGC1α (**L**), FITC-CBP/ULight-SRC1 (**M**), and FITC-TRAP220/ULight-SRC1 (**N**). The EC_50_ values and sample numbers (*n*) are indicated in parentheses. The percent maximal responses (as fold-change responses against the vehicle) of the coactivator recruitment ascertained using dual-FRET against the maximal responses determined by employing single-FRET (in **A**–**F**) are shown in bar graphs. Differences in the maximal responses are significant at ** *p* < 0.01, obtained via unpaired Student *t*-tests.

Finally, we examined all of the eight combination sets with the PPARγ-LBD activated by pioglitazone (Figure 5). FITC-CBP was preferentially recruited less than ULight-CBP (Figure 5G), but FITC-TRAP was preferentially employed more than ULight-TRAP (Figure 5J). When the maximal procurement of FITC-CBP was considered 100, those of ULight-CBP/TRAP220/PGC1α/SRC1 were 237, 107, 124, and 52.0, respectively (Figure 5G, I, K, and M, respectively). When the peak recruitment of FITC-TRAP was assumed to be 100, those of ULight-CBP/TRAP220/PGC1α/SRC1 were 295, 65.8, 154, and 57.1, respectively (Figure 5H, J, L, and N, respectively). In summary, the coactivator preference of PPARγ-LBD was considered CBP > PGC1α ≥ TRAP220 > SRC1. This is generally similar to the reverse EC_50_ orders in single-FRET; namely, CBP (0.637 µM) < PGC1α (1.07 µM) < TRAP220 (1.91 µM) < SRC1 (2.11 µM) (Figure 5C–F).

### 3.4. PPARα/γ-LBD–Corepressor Cocrystal Structures

We have revealed multiple PPARα/δ/γ-LBDs–various ligand cocrystal structures by X-ray diffraction [22,23,24,26]. All of these data (41, 4, and 6 for PPARα/δ/γ-LBDs, respectively) were registered in the PDB, which corresponds to 64.1%, 7.3%, and 1.9%, respectively, of all of the PDB deposits for PPAR as of 3/3/2025 (Table S2). Among these, a total of 13 deposits were from PPARα/γ-LBDs–various ligands–SRC1 coactivator cocrystals (blue in Table S2) but not from those with other coregulators, namely, coactivators or corepressors. Using our sophisticated cocrystallization techniques [25], we first aimed to obtain PPARα/δ/γ-LBDs–corepressor cocrystals in the absence or presence of ligands.

**Figure 6:**
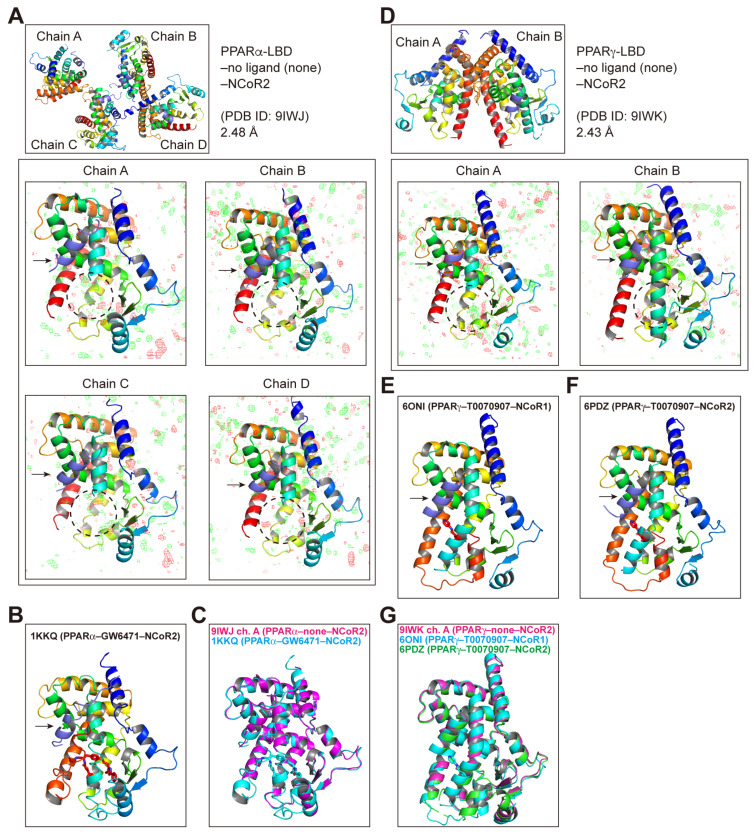
PPARα/γ-LBD–antagonist/no ligand–corepressor cocrystal structures revealed by X-ray diffraction. (**A**) The PPARα-LBD–no ligand–NCoR2 corepressor cocrystal structure obtained in this study (PDB ID: 9IWJ). The cocrystal is composed of tetramers (chains A–D), all having NCoR2 peptides (indicated by arrows) and an ambiguous (flexible) ligand-binding locus (represented by dotted circles). (**B**) The PPARα-LBD–GW6471 (PPARα antagonist)–NCoR2 corepressor cocrystal structure reported in a previous study (PDB ID: 1KKQ; Xu et al., 2002 [14]). The NCoR2 peptide is indicated by an arrow and GW6471 (in red) is surrounded by several α-helices. (**C**) The superimposed image of **A** (chain A) and **B**. GW6471 is in light blue. (**D**) The PPARγ-LBD–no ligand–NCoR2 corepressor cocrystal structure obtained in this study (PDB ID: 9IWK). The cocrystal is composed of a dimer (chains A and B), both possessing NCoR2 peptides (arrows) and an ambiguous (flexible) ligand-binding locus (dotted circles). (**E**,**F**) The PPARγ-LBD–T0070907 (PPARγ antagonist in red)–NCoR1 (**E**) or NCoR2 (**F**) corepressor (arrows) cocrystal structures determined in a previous study (PDB IDs: 6ONI and 6PDZ, respectively; Shang et al., 2020 [43]). (**G**) The superimposed image of **D** (chain A), (**E**,**F**). T0070907 is in light blue.

After screening hundreds of cocrystallization buffer sets as we had done previously [25], the PPARα-LBD–NCoR2 cocrystal was finally obtained. X-ray diffraction resolved its tetrameric structure (chain A–D) in an orthorhombic space group P212121 at 2.48Å resolution (Table S1) with an NCoR2 peptide (arrows) but not any ligand (Figure 6A), which was deposited with the PDB ID: **9IWJ**. As >80% of PPARα-LBDs in our routine preparation contained iFAs [19], the corepressor attachment likely dissociated the iFAs. Notably, the structures of the ligand-binding loci (dotted circles) were ambiguous when compared with the previously reported PPARα-LBD–GW6471 (PPARα antagonist [42])–NCoR2 cocrystals (Figure 6B) [14], whereas the other parts of PPARα-LBD were similar (Figure 6C). Next, The PPARγ-LBD–NCoR2 cocrystal was obtained and its dimeric structure was obtained in a monoclinic space group P21 at a 2.43 Å resolution (Table S1; PDB ID: **9IWK**). Its ligand-binding loci (dotted circles) were also vague (Figure 6D) compared with the PPARγ-LBD–T0070907 (PPARγ antagonist)–NCoR1/NCoR2 cocrystals [43] (Figure 6E and F, respectively), which highly contrasted with the other PPARγ-LBD structures that were similar (Figure 6G). Unfortunately, we failed to obtain the PPARδ-LBD–corepressor cocrystals.

### 3.5. PPARα-LBD–Coactivator Cocrystal Structures

**Figure 7:**
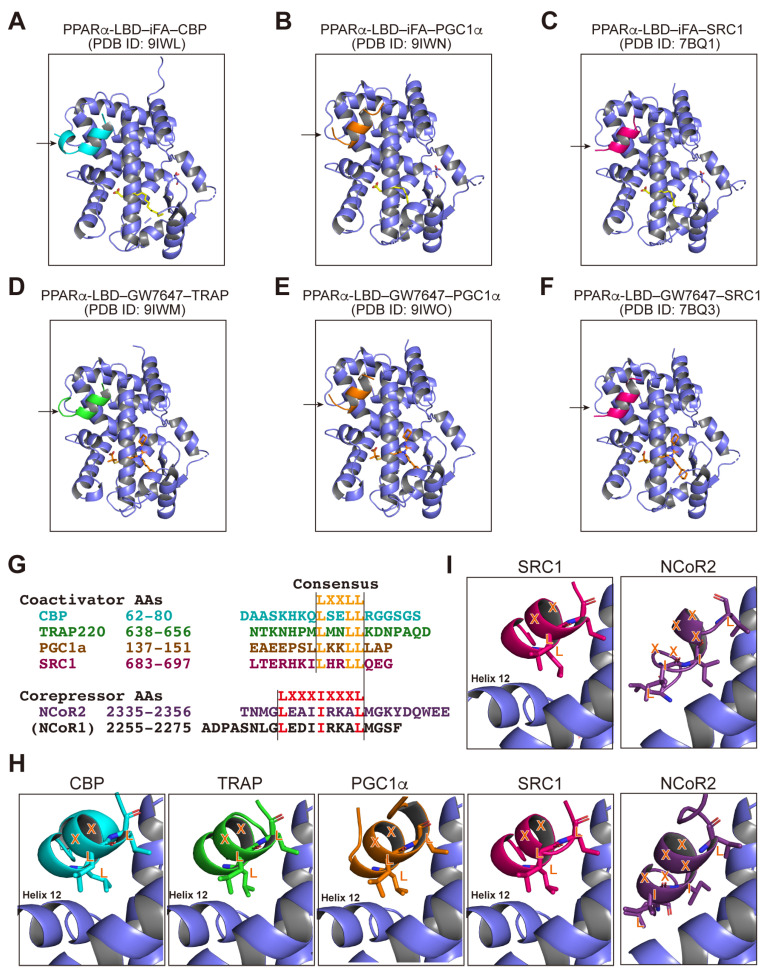
PPARα/γ-LBD–coregulator cocrystal structures revealed by X-ray diffraction. (**A**–**F**) The structures of the PPARα-LBD–iFA (**A**–**C**)/GW7647 (**D**–**F**)–CBP (**A**; PDB ID: 9IWL)/PGC1α (**B**; PDB ID: 9IWN and **E**; PDB ID: 9IWO)/SRC1 (**C**; PDB ID: 7BQ1 and **F**; PDB ID: 7BQ3 obtained from Kamata et al., 2020 [22])/TRAP220 (**D**; PDB ID: 9IWM) coactivator cocrystals obtained in this study (PDB ID: 9IWJ). Coactivator peptides are indicated by arrows. (**G**) Consensus amino acid motifs preserved in human PPAR coactivators/corepressors: LXXLL (X is any amino acid) motifs in the CBP/TRAP220/PGC1α/SRC1 coactivators and LXXXIXXXL motifs in the NCoR2(/NCoR1) corepressor. (**H**) Magnified views of the coactivator/corepressor peptides–PPARα-LBD interaction sites adopted from **A** (for CBP), **D** (for TRAP), **B** (for PGC1α), **C** (for SRC1), and Figure 6A chain A (for NCoR2). Coactivator peptides interact with the AF-2 helix 12 formed in the active (/agonist-bound) PPARα-LBD, while the NCoR2 only binds to the inactive (/antagonist-bound) PPARα-LBD that lacks the helix 12. (**I**) Magnified views of the coactivator/corepressor peptides–PPARγ-LBD interaction sites adopted from PDB ID: 8HUM (PPARγ-LBD–lanifibranor (PPAR pan agonist)–SRC1; Kamata et al., 2023 [24]) and Figure 6D chain A (PPARγ-LBD–no ligand–NCoR2). The SRC1 peptide interacts with helix 12 formed in lanifibranor-bound PPARγ-LBD, whereas the NCoR2 only binds to the inactive (/unliganded) PPARγ-LBD that lacks helix 12.

We have succeeded in obtaining 14 PPARα/γ-LBDs–SRC1 coactivator peptide cocrystals (Table S2) and aimed to obtain more with the other coactivators: CBP, TRAP220, and PGC1α. By employing crushed powders of PPARα-LBD-iFA cocrystals as crystal cores, we obtained PPARα-LBD–iFA–CBP and PPARα-LBD–iFA–PGC1α cocrystals and revealed their structures (Figure 7A and B, respectively). The iFAs bound to the same loci that were previously reported in a PPARα-LBD–iFA–SRC1 cocrystal (Figure 7C) [22]. Next, utilizing the same seeding procedures and the PPARα full agonist GW7647, we obtained the PPARα-LBD–GW7647–TRAP220 and PPARα-LBD–GW7647–PGC1α cocrystals and ascertained their structures (Figure 7D and E, respectively). GW7647 bound to the same loci as was previously reported, PPARα-LBD–GW7647–SRC1 cocrystals (Figure 7F) [22]. All four coactivator peptides contain the consensus LXXLL motifs that interact with the activation function-2 (AF-2) helix 12 upon ligand activation, whereas the two corepressor peptides contain the consensus LXXXIXXXL motifs (Figure 7G) [14]; we investigated their positions in the cocrystals. In PPARα-LBD, the α-helical LXXLL motifs in the four coactivators were located near the AF-2 helix 12 that was formed upon ligand binding, whereas the IXXXL portion of the consensus LXXXIXXXL motif in the NCoR2 coactivator was located in a position that interfered with helix 12 if present (Figure 7H). The same applied to the interaction of PPARγ-LBD and the SRC1 coactivator or the NCoR2 corepressor (Figure 7I). These structural findings support the idea that the agonist-induced formation of the AF-2 helix 12 induces the dissociation of the corepressors and the association of the coactivators.

## 4. Discussion

To date, hundreds of NR coregulators (coactivators and corepressors) with various types of structures and functions have been identified [44]. These coregulators are differentially expressed in various cells/tissues and have altered physiological functions, as most clearly evidenced by knockout studies in mice [10,11,12,17,45,46]. They are likely shared by a total of 48 NRs in humans and could be the “Servants and Masters for Control of Systems Metabolism” [46] of the NRs. For example, the respective PPARα/δ/γ agonists: pemafibrate, seladelpar, and pioglitazone could recruit at least four coactivators—CBP, TRAP220, PGC1α, and SRC1—to PPARα/δ/γ-LBDs with altered efficacy and efficiency, although each PPARα/δ/γ-LBD can accept only one coactivator directly via interaction between its LXXLL motif and the newly formed AF-2 helix 12 upon agonist binding [21]. Therefore, various PPAR agonists, including endogenous fatty acids [22], could differentially induce gene expression via the same PPAR-LBD–PPRE system, depending on the expression patterns of coactivators within each cell. Furthermore, some coactivator members within the multiprotein coactivator complex have several LXXLL motifs (e.g., human CBP, SRC1, and TRAP220 contain 2, 7, and 2 LXXLL motifs, respectively) and may bind to both PPAR and RXR in different ways [28]. PPAR coactivators demonstrate varying intrinsic biological activities, including some enzymes, such as histone acetylases (e.g., CBP) and ATPases, responsible for chromatin remodeling, and proteins functioning in between the NRs and the transcription initiation machinery, such as TRAP220, PGC1α, and SRC1 [17,20,21,27,28]. In another example, tamoxifen, a selective estrogen receptor (ER) modulator used to treat breast cancer, acts as either an ER agonist or antagonist depending on cancer cell types, probably due to the altered relative expressions of coactivators and corepressors [47]. Thus, an understanding of the coregulator dynamics of the NR-LBDs is very vital, from a pharmacological point of view.

In this study, dual-FRET technology was employed for the simultaneous detection of corepressor dissociation/coactivator recruitment (Figure 2M–R) and competitive coactivator binding (Figure 3, Figure 4 and Figure 5) with the PPARα/δ/γ-LBDs, utilizing the Tb donor and two fluorescence (FITC and ULight) acceptors within a single well of a 384-well microplate. Dual FRET revealed the coactivator preferences of PPARα/δ/γ-LBDs, which were generally similar to the reverse EC_50_ orders obtained using single-FRET (Figure 3, Figure 4 and Figure 5). This method also allows the extremely sensitive detection of the NR agonist, antagonist, and inverse agonist activities [48], the interactions between PPAR/RXR/thyroid hormone response element (TRE) [49], and the ER/coactivator/ER ligand [50]. Furthermore, an optically multiplexed, six-color FRET assay was developed to detect five different individual binding events [51], even though the “antenna effect” and “FRET surplus” may affect such multiplexed FRET efficiency [52], as was observed with the varied activities of FITC-CBP and ULight-CBP (Figure 3G, Figure 4G and Figure 5G). Overcoming them would enable the simultaneous detection of the interactions between the NR and the other five molecules, such as RXR, coactivators, corepressors, and DNA-responsive elements in the future. If this is the fundamental limitation of dual/multiplexed FRET assay, other biochemical methods such as surface plasmon resonance assay may be favorable for direct binding affinity determination if there is no sensitivity issue.

In this study, we obtained for the first time, two PPARα/γ-LBD structures complexed with the corepressor but without any ligand (PDB ID: **9IWJ** and **9IWK**; Figure 6A and D, respectively; Table S2). Electron density of the amino-terminus (AAs 257–279) of helix 3 and the carboxy-terminus of helix 11 to helix 12 (AAs 448–468) was lacking in all four chains of the PPARα-LBD (Figure S1A), which contrasts with the structures of the PPARα-LBD–PPARα antagonist GW6471–NCoR2 corepressor (PDB ID: **1KKQ** [14]; Figure S1B) as well as the PPARα-LBD–PPARα agonist GW7647–SRC1 coactivator (PDB ID: **7BQ3** [22]; Figure S1C). Similarly, in chain A of PPARγ-LBD, the electron density of the amino-terminus (AAs 260–288) of helix 3 and the carboxy-terminus of helix 11 to helix 12 (AAs 457–477) was lacking (Figure S1D), which also contrasts with the structures of the PPARγ-LBD–PPARγ inverse agonist T0070907–NCoR2 (PDB ID: **6PDZ** [43]; Figure S1E) as well as that of PPARγ-LBD–PPARα/δ/γ pan agonist lanifibranor–SRC1 (PDB ID: **8HUM** [24]; Figure S1F). Therefore, antagonist/agonist binding may stabilize these α-helical structures. Notably, PPARγ inverse agonists (T0070907 and SR10171) stabilize the AF-2 helix 12 at alternative locations (Figure 5E and Figure S1E for T0070907) [43,47] while no ligand (Figure S1A,D) or antagonist binding (Figure S1B) form at helix 12 at any location, both of which facilitate corepressor recruitment (Figure 7H,I).

The enhanced degree of structural flexibility of the amino-terminus of helix 3 and the carboxy-terminus of helix 11 to helix 12 of both unliganded PPARα/γ-LBDs (Figure 6A,D) was supported by two independent solution-phase amide hydrogen/deuterium exchange (HDX) analyses that revealed stabilization of the PPARγ-LBD AAs 279–287 (helix 3) and AAs 470–477 (helix 12) by rosiglitazone [53,54]. Many previous studies have mentioned that PPAR ligands enter the Y-shaped ligand-binding portion of the PPARs through a small opening named the “entrance”, composed of polar residues such as Pro227, Arg288, Glu295, and Glu343 in PPARγ [55]. However, our results suggest that any ligand can enter this large ligand-binding cavity [22] through these flexible regions rather than the entrance identified in the agonist-bound PPARγ-LBD cocrystal structures. The ligand activities might depend on the stability of their existence within the structures.

## 5. Conclusions

We have demonstrated coregulator dynamics (coactivator replacements with corepressor and coactivator competitions) on the PPARα/δ/γ-LBDs employing a dual-FRET assay. We also revealed the structures of the unliganded PPARα/γ-LBD–NCoR2 corepressor and PPARα-LBD–CBP/TRAP220/PGC1α (/SRC1; [19]) coactivator cocrystals utilizing X-ray diffraction. These findings provide the molecular basis for the mechanisms by which the coactivator replaces the corepressor and the coactivators compete for the very limited surfaces of PPARα/δ/γ-LBDs. Until now, PPAR drug discovery has focused almost entirely on the PPAR subtype selectivity of each ligand. However, this study proposes the idea that how/which corepressors keep PPAR signals off in the steady-state condition and how/which coactivators are recruited to drive specific gene transcription upon ligand binding in particular cell types (especially, PPAR target cells in clinical settings such as cancer cells) are also critical. Differential transcriptional controls via different coactivator recruitments induced by different PPAR subtype/agonist sets should next be investigated at the cell level.

## Data Availability

All of the data is contained within the article and the Supplementary Materials. The X-ray diffraction datasets have been deposited in the PDB with accession numbers: **9IWJ**, **9IWK**, **9IWL**, **9IWM**, **9IWN**, and **9IWO**.

## Supplementary Materials

The following supplementary materials can be downloaded at https://www.mdpi.com/article/10.3390/antiox14040494/s1, Table S1: Data collection and refinement statistics (molecular replacement); Table S2: PPAR–coregulator cocrystal structures deposited in the Protein Data Bank (as of 3/3/2025); Figure S1: PPARα/γ-LBD–coregulator cocrystal structures with/without ligands.
