# Supplementary material for: Competitive Ligand-Induced Recruitment of Coactivators to Specific PPARα/δ/γ Ligand-Binding Domains Revealed by Dual-Emission FRET and X-Ray Diffraction of Cocrystals
Source: Antioxidants (Basel). 2025 Apr 20;14(4):494. doi: 10.3390/antiox14040494 (PMC12024163; doi:10.3390/antiox14040494)
Supplement: Supplementary file 1 [file antioxidants-14-00494-s001.zip › antioxidants-3538152-supplementary.pdf]

Supplementary Materials

Competitive Ligand-Induced Recruitment of Coactivators to Specific PPARα/δ/γ Ligand-Binding Domains Revealed by Dual-Emission FRET and X-Ray Diffraction of Cocrystals

Contents:

Table S1. Data collection and refinement statistics (molecular replacement)

Table S2. PPAR–coregulator cocrystal structures deposited in the Protein Data Bank (as of 3/3/2025)

Figure S1. PPARα/γ-LBD–coregulator cocrystal structures with/without ligands

Table S1. Data collection and refinement statistics (molecular replacement)

Protein	hPPARα-LBD	hPPARγ-LBD	hPPARα-LBD	hPPARα-LBD	hPPARα-LBD	hPPARα-LBD
Peptide	NCoR2	NCoR2	CBP	TRAP220	PGC1α	PGC1α
Binding ligand	none	none	iFA	GW7647	iFA	GW7647
PDB ID	9IWJ	9IWK	9IWL	9IWM	9IWN	9IWO
Data collection						
Space group	$P2_12_12_1$	$P2_1$	$P2_1$	$P2_1$	$P2_1$	$P2_1$
Cell dimensions						
a, *b*, *c* (Å)	99.73, 112.15, 122.82	61.30, 60.05, 92.83	44.91, 60.99, 53.95	45.01, 61.92, 52.94	45.03, 61.50, 53.27	45.02, 62.20, 53.31
α, β, γ (°)	90.00, 90.00, 90.00	90.00, 105.66, 90.00	90.00, 110.53, 90.0 0	90.00, 106.61, 90.00	90.00, 106.29, 90.00	90.00, 106.16, 90.00
Resolution (Å)	48.88–2.48 (2.56–2.48)	44.69–2.43 (2.52–2.43)	42.06–2.09 (2.15–2.09)	43.13–1.39 (1.41–1.39)	43.22–1.59 (1.62–1.59)	43.24–1.49 (1.52–1.49)
R_{merge}	0.036 (0.362)	0.038 (0.398)	0.046 (0.385)	0.035 (0.341)	0.052 (0.358)	0.033 (0.343)
R_{pim}	0.015 (0.150)	0.024 (0.244)	0.030 (0.248)	0.022 (0.213)	0.033 (0.224)	0.021 (0.154)
$CC_{1/2}$	1.000 (0.947)	0.999 (0.897)	0.999 (0.843)	0.999 (0.924)	0.998 (0.874)	1.000 (0.233)
$I / \sigma I$	28.9 (4.9)	16.1 (2.8)	21.6 (3.9)	17.1 (3.2)	14.0 (3.2)	17.6 (3.0)
Completeness (%)	100.0 (99.9)	98.7 (97.7)	99.1 (98.4)	97.5 (95.7)	99.8 (99.2)	99.7 (99.3)
Redundancy	6.6 (6.7)	3.5 (3.5)	3.3 (3.3)	6.6 (6.7)	3.5 (3.5)	3.3 (3.3)
**Refinement**						
Resolution (Å)	38.711–2.480	44.084–2.430	27.874–2.090	43.130–1.390	43.219–1.590	43.240–1.490
No. reflections	49,439	24,381	16,128	54,640	37,468	46,018
R_{work} / R_{free}	0.2249/0.2543	0.2382/0.2655	0.1780/0.2357	0.1835/0.2048	0.1860/0.2081	0.1938/0.2135
No. atoms						
Protein	7,495	3,881	2,186	2,232	2,207	2,187
Ligand	0	0	24	41	24	35
Water	0	0	34	146	109	101
B-factors	63.37	67.06	37.36	19.69	19.16	25.10
Protein	63.37	67.06	37.37	19.34	18.91	25.04
Ligand			45.62	19.57	29.02	21.19
Water			30.58	25.12	21.93	27.73
Ramachandran plot (%)						
Favored	95.80	96.24	95.85	97.41	97.73	98.12
Allowed	3.12	3.34	3.40	1.85	2.27	1.13
Outliers	1.08	0.42	0.75	0.74	0.00	0.75
R.m.s. deviations						
Bond lengths (Å)	0.002	0.002	0.010	0.014	0.010	0.011
Bond angles (°)	0.52	0.41	1.11	1.32	1.05	1.05

iFA, intrinsic fatty acid. Values in parentheses are for highest-resolution shell.

Table S2. PPAR–coregulator cocrystal structures deposited in the Protein Data Bank (as of 3/3/2025)

Subtypes	Total PDB deposits [Ours]	Deposits with coregulators [Ours]	Deposits with coactivators					Deposits with corepressors		
			CBP	TRAP220	PGC1alpha	SRC1	others	NCoR1	NCoR2	others
PPAR alpha	64 [46]	27 [14]	9IWL (iFA)	9IWM (GW7647)	9IWN (iFA)<br>9IWO (GW7647)<br>6KXY, 6KXX	8HUK (lanifibranor)<br>8HUQ (elafibranor)<br>7BPY (clofibric acid)<br>7BPZ (bezafibrate)<br>7BQ4 (EPA)<br>7BQ3 (GW7647)<br>7BQ2 (pemafibrate)<br>7BQ1 (iFA)<br>7BQ0 (fenofibric acid)<br>7C6Q, 6L96, 5AZT, 3FEI, 3G8I, 3ET1, 2NPA, 2P54, 1K7L	3SP6	0	9IWJ (no ligand)<br>1KKQ (GW6471)	0
PPAR delta	55 [4]	0 [0]	0	0	0	0	0	0	0	0
PPAR gamma	316 [7]	112 [6]	7RLE, 1RDT	6ONJ, 6DGP, 6D94	9F7W, 9F7X, 8DK4, 8BF1, 6T1V, 6MS7, 6IZM, 6IZN, 6FZF, 6FZJ, 6FZP, 6AD9, 5Z5S, 5Z6S, 5TWO, 3V9T, 3V9V, 3U9Q, 3B1M, 3CS8	8HUM (lanifibranor)<br>8HUP (seladelpar)<br>7WGO (bezafibrate)<br>7WGP (fenofibric acid)<br>7WGQ (pemafibrate)<br>7CXF, 7CXH, 7CXI, 7CXJ, 7CXK, 7CXL, 6KTM, 6KTN, 6K0T, 6IJR, 6IJS, 6JQ7, 6ICJ, 6ILQ, 5YCN, 5YCP, 5GTN, 5GTO, 5GTP, 5JI0, 5DSH, 5DV3, 5DV6, 5DV8, 5DVC, 5DWL, 4Y29, 4HEE, 4FGY, 4F9M, 3S9S, 3T03, 3VN2, 3QT0, 3V9Y, 3LMP, 3KMG, 3FEJ, 3FUR, 3H0A, 3G9E, 3ET3, 3CWD, 2GTK, 2HFP, 2FVJ, 1K74, 1FM6, 1FM9, 2PRG	9CK0, 3DZU, 3DZY, 3E00, 1ZGY, 1WM0	8FKC, 8FKD, 8FKE, 8FKF, 8FKG, 8FHE, 8FHG, 8DKN, 8DKV<br>6ONI (T0070907)	9IWK (no ligand)<br>8B8W, 8B8X, 8B8Y, 8B8Z, 8B90, 8B91, 8B92, 8B93, 8B94, 8B95, 8AQM, 8AQN, 7SQA<br>6PDZ (T0070907)	9CWN

The cocrystal structures reported in this study and our previous studies are labelled in red and blue, respectively.

The structures referred in this study are labelled in green.

The underlined structures are web-linked to the PDB homepage.

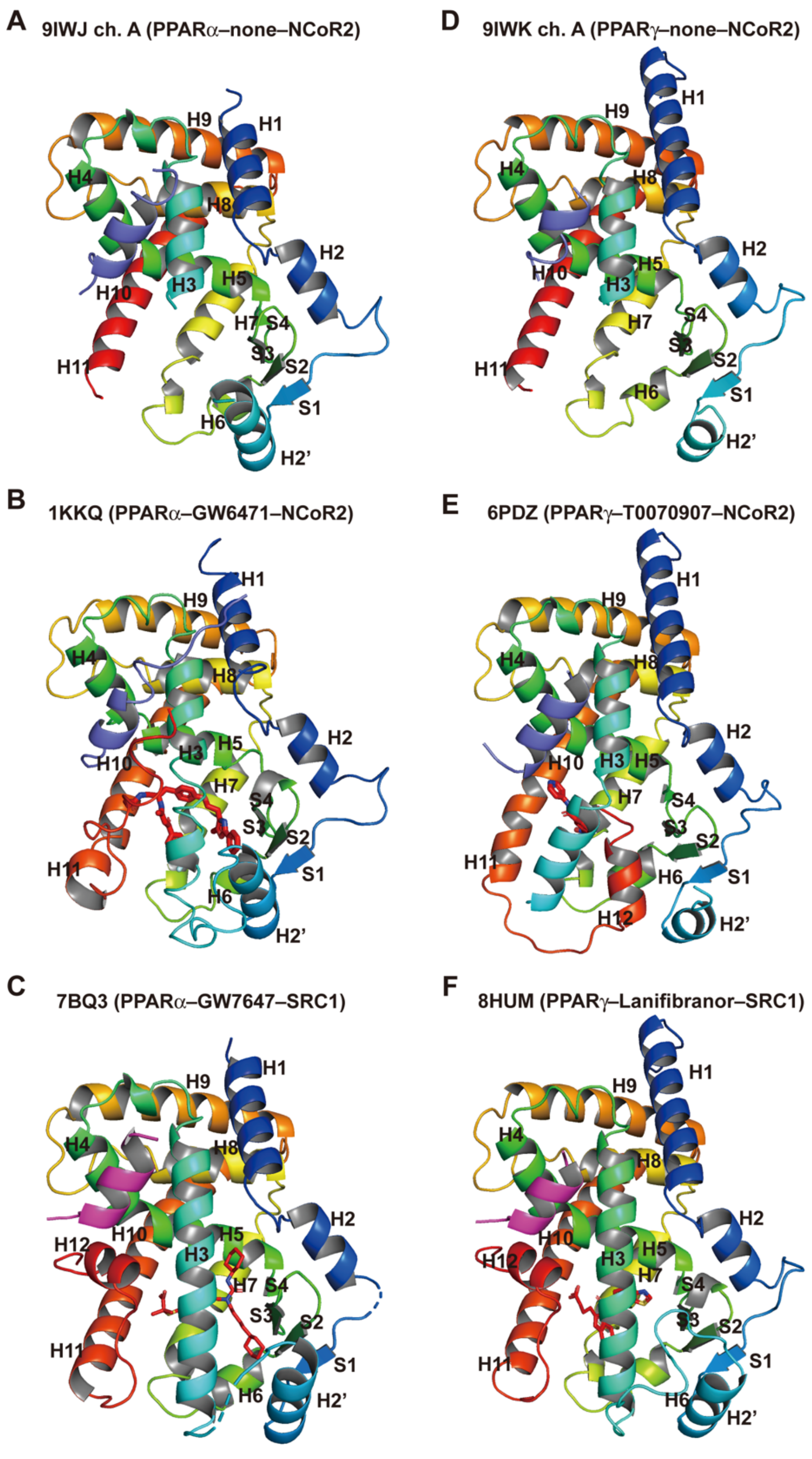

Figure S1. PPARα/γ-LBD–coregulator cocrystal structures with/without ligands. (**A**–**C**) PPARα-LBD–coregulator structures with no ligand (A), antagonist (GW6471; B), and agonist (GW7647; C). (**D**–**F**) PPARγ-LBD–coregulator structures with no ligand (D), inverse agonist (T0070907; E), and agonist (lanifibranor; F). The numbers of α-helix (H) and β-sheet (S) structures, PDB IDs, and associated coregulators (NCoR2 corepressor or SRC1 coactivator) are indicated.
